# Association of Gastrointestinal Symptoms and Psychological Issues With the Intake of Fruits, Vegetables, and Junk Food in School Children: A Community-Based Study From Melmaruvathur, Tamil Nadu, India

**DOI:** 10.7759/cureus.104262

**Published:** 2026-02-25

**Authors:** Prasanna Venkatesh, Padma K, Raahavendhar Sugumar, Sivagamasundari Venugopal

**Affiliations:** 1 Paediatrics, Melmaruvathur Adhiparasakthi Institute of Medical Sciences and Research, Melmaruvathur, IND

**Keywords:** fruits and vegetables, gastrointestinal symptoms, junk food, nutrition, psychological issues, schoolchildren

## Abstract

Background

Gastrointestinal (GI) symptoms are among the most common and rapidly increasing symptoms in pediatric outpatient practice. Similarly, psychological issues and abnormal nutritional status are also worsening in school-going children. GI morbidity, psychological issues, and nutrition are intricately related to diet, especially fruit and vegetable (F&V) and junk food intake. This study aimed to explore the current prevalence of significant GI symptoms, psychological issues, and nutrition in schoolchildren and their association with F&V and junk food intake.

Methodology

This was a cross-sectional study conducted among schoolchildren in classes 1-10 from a semi-rural private school. A prevalidated questionnaire for common GI symptoms and their severity was completed by 394 students. A semi-quantitative focused Food Frequency Questionnaire (FFQ) was used for F&V and junk food intake. Psychological screening was done using the Strengths and Difficulties Questionnaire (SDQ). Body mass index (BMI) was obtained from school records. Microsoft Excel and SPSS version 29 were used for descriptive statistics, and Mann-Whitney U tests with effect sizes were used for non-parametric analysis.

Results

Significant GI symptoms were present in 113/394 (28.7%) children. SDQ screening revealed that psychological issues were probable in 66/354 (18.6%) and definite in 58 (16.4%) students. BMI was abnormal in 149/385 (38%), with thinness noted in 43 (11.3%), severe thinness in 32 (8.3%), overweight in 53 (13.7%), and obesity in 21 (5.55%) children. Median greens intake was 6.43 g/day, at a median frequency of one to two times per week, with 74.4% of the children consuming less than 25% of the recommended intake. Median intake of vegetables was 30.5 g/day, at a frequency of one to two times per day, with 83.3% of children consuming less than 25% of the recommended intake. Fruit intake was 40 g/day, at a frequency of three to four times per week, with 45% of the children consuming 50-75% of the recommended intake. Total median F&V intake was 77.4 g/day, at a frequency of one to two times per day, with 63% consuming less than 25% of the recommended intake, and a mere 1.8% consumed 100% or more of the recommended intake. The median frequency of junk food intake was 2.3 times per day. Common junk foods included biscuits, health drinks, and home-made oily, high-calorie foods. Junk food intake was associated with statistically significant, adverse GI, psychological, and nutritional outcomes. F&V intake did not show a statistically significant association with any of the above parameters.

Conclusions

Significant GI symptoms are very common in schoolchildren. Nutritional indices are worsening, and psychological issues are also not uncommon in school children. Junk food intake is very high and increasing alarmingly, and is significantly associated with adverse GI, psychological, and nutritional outcomes in schoolchildren. F&V intake is very poor and is decreasing more than ever before in schoolchildren. These findings are concerning. Suitable action must be taken on a war footing at the individual, family, school, and governmental levels to increase F&V intake and decrease junk food intake in schoolchildren to reduce both immediate morbidity and ensure the future health of children. This can be done through greater awareness among parents, children, teachers, and governmental commitment to improve gut health, psychological health, and nutrition of schoolchildren.

## Introduction

Gastrointestinal (GI) symptoms are among the most common symptoms noted in school-age children, and their prevalence is increasing rapidly [1]. Intuitively and by scientific research, GI symptoms have been linked to diet, especially the intake of junk food [2]. Many studies have established the adverse role of junk food, especially ultra-processed foods, in common GI illnesses such as inflammatory bowel disorders and functional gastrointestinal disorders (FGIDs). Moreover, several studies have comprehensively demonstrated the protective role of fruits and vegetables (F&V) not only in GI disorders but also in most chronic lifestyle diseases, including diabetes, hypertension, cancer, and psychological issues.

Due to a rapid “nutrition transition,” intake of ultra-processed junk food is exploding, with its palatability and addictive nature rapidly eroding the intake of protective foods such as F&V. This has led not only to the increasing triple burden of malnutrition, namely, underweight, obesity, and micronutrient deficiency [3,4], but also the increasing prevalence of adverse mental health in schoolchildren.

Although tremendous strides have been made in the treatment of various complex childhood illnesses across diverse fields, the nutritional and mental well‑being of children, the very foundation of good health, appears to be rapidly deteriorating. Numerous studies are available on the role of diet in GI diseases, but few community-based studies have been conducted in India on the impact of F&V and junk food on various GI symptoms [5], as well as nutritional and psychological issues. Hence, we aimed to estimate the prevalence of (i) significant recurrent GI symptoms (weekly for ≥2 months), (ii) psychological distress (the Strengths and Difficulties Questionnaire (SDQ)), and (iii) abnormal body mass index (BMI) (assessed using the World Health Organization classification), and to examine their associations with F&V intake and junk food consumption among schoolchildren in semi-rural Tamil Nadu.

## Materials and methods

Study design and setting

A cross-sectional analytical study was conducted among students from classes 1-10 of a private school in a semi-rural area in Tamil Nadu, India, from November 2022 to February 2023.

Sample size calculation

A sample size of 288 was calculated using the following formula: n = (Z² × p × (1 − p))/d², where n is the required sample size, Z is the Z-value for the desired confidence level (1.96 for 95% confidence interval (CI)), p is the expected prevalence (proportion), d is the desired absolute precision (margin of error) expected margin of error (0.05).

In their study, Robin et al. identified an FGID prevalence of 25%. This was taken as a surrogate prevalence of significant GI symptoms [6]. FGID prevalence was used because it is a predominantly symptom-based diagnosis based on ROME IV criteria [7], where children have significant recurring GI symptoms such as pain, bloating, and constipation, which significantly impact their day-to-day activities. A 10% allowance for nonresponse was taken, and the final sample size arrived at was 317. The students were invited to participate by announcing in their assembly, respective classrooms, parent-teacher meets, and through messages in their respective class WhatsApp groups. Periodic reminders were sent until an adequate sample size was obtained. Convenience sampling was done. All healthy children from classes 1-10 who consented were enrolled in the study.

Inclusion and exclusion criteria

Children with major chronic illness which could cause significant GI symptoms such as inflammatory bowel diseases, epilepsy on chronic medication, long-standing lung and cardiac illnesses such as cystic fibrosis and congestive cardiac failure, and children with major developmental and neurologic disorders that impact dietary intake were excluded. Children on long-term medication for any illness were excluded, as these medicines can cause GI symptoms.

Ethical clearance

After obtaining Institutional Ethical Committee clearance (approval number: MAPIMS/IEC/52/2023) and due consent and assent from participants, data were collected from mothers of children from classes 1-5 over the phone and directly from students from classes 6-10.

Data collection

Doctors (at least MBBS) were pre-trained to ensure uniformity in collecting data. The data collectors were carefully instructed about how to gather data, and each was given a sample of five children of different ages for data collection during the pilot study, and monitored and corrected as required to ensure inter-rater fidelity in data collection. The same questionnaire was employed for both mothers of children from classes 1-5, over the phone, and directly for older children for the questionnaire on GI symptoms and diet. SDQ was administered in two age groups as per the recommendations of the developers of the instrument.

Questionnaire design

The study questionnaire (Appendices) was designed after an extensive review of previously published literature and relevant guidelines. Psychological distress was assessed using the SDQ, an established and internationally validated behavioral screening tool. Dietary intake, including total junk food consumption (composite score) and green F&V grams per day, was measured via a focused Food Frequency Questionnaire (FFQ), with content validation conducted through expert review by five registered dietitians to ensure item relevance for Indian pediatric populations (content validity index >0.80). GI symptoms were evaluated using a single binary item criterion, referenced to the Rome IV diagnostic criteria, while BMI category was determined via standardized calculations based on WHO age- and sex-specific protocols. Each item was evaluated for clarity, relevance, and comprehensiveness by senior pediatricians, and due changes were made based on expert feedback to ensure adequate domain coverage. A small pilot study was conducted in another school, consisting of about 30 students, and the questionnaire was modified as appropriate. Data from the pilot study were not included in the final analysis.

Data collection instrument, including GI symptoms and FFQ, was interviewer-administered and consisted of the below parts.

Demographic Details

Demographic data included name, age, sex, education, and profession of both parents.

GI Symptoms

The students were queried about 11 common GI symptoms usually noted in children, including abdominal pain, diarrhea, vomiting, constipation, nausea, early satiety, bloating, rumination/regurgitation, indigestion, aerophagy, and fecal incontinence over the previous three months from the time of applying the questionnaire. Symptoms at least once a week, for more than two months, were categorized as significant symptoms. Symptoms fewer than once per week and/or fewer than two months were classified as occasional symptoms. Among students with significant symptoms, the duration of the problem and the frequency and severity of the symptoms were further elicited.

Dietary Intake

**Figure 1:**
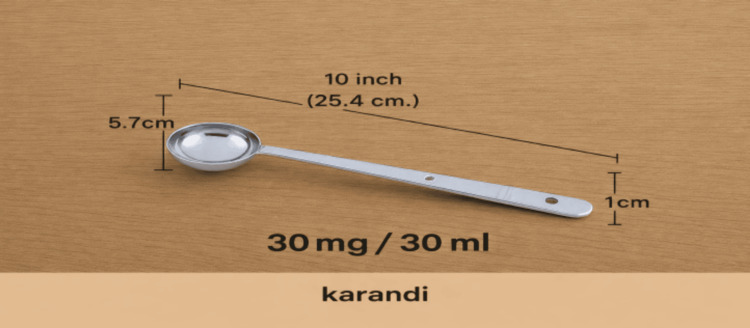
Karandi (ladle).

A focused semi-quantitative FFQ was administered, with details of intake of fruits, vegetables, greens, and junk food consumption. Students were asked about their intake on any average day. If consumption was fewer than once per day, the intake in a regular week or month was queried. Portion sizes for vegetables were calculated in terms of “karandi” or a ladle of approximate volume of 30 mL/30mg (Table 1). A karandi (Figure 1) is the usual measure used to serve vegetables and other foods in a normal household in Tamil Nadu. The image of the karandi with specific dimensions was shown to the interviewee over WhatsApp for the phone interview and shown directly to students during direct questioning. Intake was averaged out as g/day and frequency/day. Portion sizes for fruits were quantified as presented in Table 1. Fruit juices were not considered a serving of fruit.

**Table 1:** Serving size descriptions and local portion equivalents.

Category	Food item	Standard serving size (80 g)	Local portion equivalent	Notes
Fruits	Apple (medium)	1 medium fruit (80 g)	1 medium fruit (80 g)	—
	Banana (medium)	1 medium fruit (80 g)	1 medium fruit (80 g)	—
	Papaya	80 g	~4–5 heaped tablespoons	1 tbsp ≈ 15–20 g
	Grapes	16 grapes (80 g)	16 grapes	Average grape size
	Plums	2 medium (80 g)	2 medium (80 g)	—
	Strawberries	6 pieces (80 g)	6 pieces	—
	Orange/Mosambi	1 medium fruit (80 g)	1 medium fruit (80 g)	—
Vegetables	Raw vegetables	80 g	~3 medium karandis	1 karandi ≈ 30 g
	Cooked vegetables	80 g	~2 medium karandis	Reduced volume after cooking
	Cooked greens	50 g	~1.5 medium karandis	Counted as vegetables
Mixed vegetables	Mixed vegetable curry	80 g	~2 medium karandis	Only the approximate amount of vegetables in the curry was documented

The WHO recommendations for minimum F&V intake in children over nine years old are 400 g and 350 g in children aged between five and nine years [8], consisting of five servings of 80 g of F&V per day [9], including greens but excluding tubers. When F&Vs are considered individually, the standard recommended intake is three servings of vegetables, two servings of fruit [10]. As the WHO has no specific recommendations for greens alone, the ICMR recommendations of 50 g/day, was considered when the adequacy of cooked greens was evaluated [11].

A list of commonly preferred junk foods among the students was identified in the pilot study and classified into (1) biscuits; (2) chocolates; (3) ultraprocessed health drinks like Horlicks, Complan, Pediasure, etc.; (4) Indian sweets such as ladoo; (5) packaged chips and similar foods; (6) cool drinks, which included all sugar-sweetened beverages and packaged juices but did not include fresh fruit juice; (7) icecream; (8) oily hotel food such as biriyani and fried rice; (9) fast food such as samosas, puff, instant noodles, pizza and pasta, and deep-fried savories; (10) bakery foods such as bread, bun, and cakes; and (11) homemade high-calorie foods such as biriyani, fried rice, and deep-fried savories. Apart from consuming high-calorie foods while eating out, food cooked at home is changing fast from traditional low-oil, healthy cooking to high-calorie, oily foods due to their high palatability. Mothers frequently cook these foods as children like them and habituate children to high-calorie foods. To explore how frequently high-fat and calorie-dense foods are consumed at home, we created a separate category of homemade junk. This aspect of junk food has not been explored in previous studies, to our knowledge.

Only the frequency of intake per day was noted for junk food consumption. The consumption of junk food was very varied in the type and amount of foods consumed and the number of times per day, with branded and unbranded foods of differing quantities. It was procured from petty shops, roadside vendors, and different hotels with varying quality and portion sizes. Similarly, even homemade junk food intake was not uniform in terms of calories and portion sizes. Therefore, quantifying junk food was nearly impossible. Moreover, the adverse effects of junk food are not merely due to calories alone but also due to the sugar, salt, oil, and other factors, such as chemicals used in processing, and whether it belongs to the ultraprocessed category. Therefore, we decided to analyze only how frequently junk food was consumed in a day.

Psychological Screening

Using the SDQ [12], children were classified as having no, possible, or definite issues. SDQ for psychological issues was applied as per the recommendations of the developers of the instrument, age-wise in two groups of 4-10 years and 11-17 years. It was filled out by parents (for children up to 10 years), by students themselves for children 11 years and above, and by teachers for all students. In the SDQ, both parent/student reports and teacher reports were collected from all participants, and both were used to obtain the total SDQ score. According to the official SDQ website maintained by the copyright holder, Prof. Robert Goodman, no written permission is required for non-commercial academic research, and it was only administered as a whole and not in parts, without any modification or translation.

Nutrition

BMI data were curated from school records and classified, as per the field tables of WHO [13].

Statistical analysis

Data was analyzed using Excel and SPSS Statistics for Windows, Version 29.0 (IBM Corp., Armonk, NY, USA). Categorical variables were expressed as frequencies and percentages, and continuous variables were summarized using mean, median, and interquartile range (IQR). Normality was assessed before inferential analysis. As several variables were non-normally distributed, non-parametric tests were used. Group comparisons for continuous variables were performed using Mann-Whitney U tests (two outcomes × three groupings; m = 6) with effect size estimation. Effect sizes: rank-biserial r = |Z| / √N (small: ~0.1, medium: ~0.3). Missing data were minimal (<10% across variables) and handled via list-wise deletion to preserve data integrity without imputation, as patterns were non-systematic. To account for multiple comparisons, Bonferroni correction was applied (family-wise α = 0.05/6≈0.008), with raw p-values reported for transparency and adjusted significance denoted. A p-value <0.05 was considered statistically significant.

## Results

Demographic characteristics

Of the 1,000 students invited to participate, 394 consented, with 73 from classes 1-5 and 321 from classes 6-10. In total, 394 students completed the proforma on GI symptoms and dietary intake, 354 completed SDQ, and BMI data were available for 385. Overall, 203/394 (51.5%) were girls. The mean age of girls was 11.6, and that of boys was 11.3 years. Further, 50% of parents were at least graduates. Most fathers were skilled workers or professionals (78.4%), and most mothers were homemakers (68%). Data was gathered from parents for 73 children from classes 1 to 5. The rest of the questionnaires were filled out by doctors after enquiring the students directly.

Gastrointestinal symptoms

**Figure 2:**
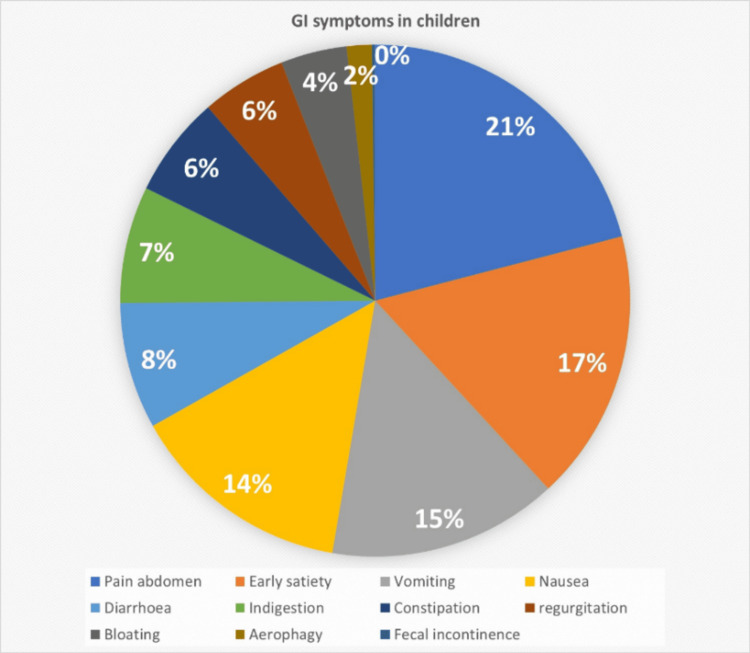
Most common symptoms noted in the students.

In total, 191 (48.4%) students recalled no GI symptoms over the past three months, and 203 (51.5%) recalled at least one. Of these, 44% had occasional symptoms (<10 times in the last three months, usually <2-3), 2.5% had weekly symptoms, but lasting fewer than two months, and 113 (53.2%) had significant symptoms at least weekly, for more than two months. The most common symptoms were abdominal pain (105, 26.7%), early satiety (86, 21.8%), vomiting (73, 18.53%), nausea (71, 18.02%), diarrhea (40, 10.15%), and constipation (32, 8.12%) (Figure 2).

Of the 113 children with significant symptoms, 34 (30.1%) had symptoms <6 months, 24 (21.2%) for up to one year, 12 (10.6%) for up to two years, and 43 (38%) for more than two years. Some had symptoms since early childhood, with the longest being eight years.

Psychological issues

Complete parent/student and teacher reports were available for 354 students, of which 69 were parent-reported (class 1-5), and 285 were student reports; teacher reports were available for all. As per the SDQ, 230 of 354 (65%) children had no psychological issues. Overall, 66 (18.6%) had possible and 58 (16.4%) had definite issues (Table 2).

**Table 2:** Psychological issues and BMI in schoolchildren. SDQ = Strengths and Difficulties Questionnaire; BMI = body mass index

Serial number	Variable	Boys, N (%)	Girls, N (%)	Total, N (%)
1. SQD (total 354)				
a	Normal	110 (47.8)	120 (52.1)	230 (65)
b	Possible psychological issue	30 (45.5)	36 (54.5)	66 (18.6)
c	Definite psychological issue	30 (8.5)	28 (7.9)	58 (16.4)
2. BMI (total 385)				
a	Normal	97 (25.2)	139 (36.1)	236 (61.3)
b	Abnormal	88 (22.9)	61 (15.8)	149 (38.7)
	Severe thinness	22 (5.7)	10 (2.6)	32 (8.3)
	Thinness	23 (6)	20 (5.2)	43 (11.2)
	Overweight	28 (7.3)	25 (6.5)	53 (13.8)
	Obese	15 (3.9)	6 (3.9)	21 (5.5)

Nutritional parameters

BMI of 236 of 385 (61.3%) was normal, 149 (38.7%) was abnormal, with thinness noted in 43 (11.3%), severe thinness in 32 (8.3%), overweight in 53 (13.7%), and obesity in 21 (5.55%) (Table 2).

Fruits, vegetables, and junk food intake

Intake of greens, vegetables, fruits, and junk food is represented in Table 3 and Table 4. Median greens intake was 6.43 g/day, 74.4% consumed less than 25% of the recommendations, and only 1.8% met the recommendations. Median vegetable intakem including greens (excluding tubers), was 30.5 g/day. Overall, 83.3% consumed less than 25% of the recommended amount, 3% met 100% of the recommended amount. Median fruit intake was 40 g/day. Overall, 45% consumed 50-75% and 43% consumed 75-100% of the recommendations, with none meeting 100%. Considering F&V together, the median intake was 77.4 g/day, 63% were consuming less than 25%, and only 1.8% were consuming 100% or more of the WHO recommendations. Overall, the median frequency for greens was one to two times per week. For vegetables, one to two times per day, and for fruits, three to four times per week. All three considered together, the intake was one to two times per day.

**Table 3:** Frequency and quantity of intake of greens, fruits, vegetables and junk food. Frequency: Assigned daily score: never = 0, monthly once = 0.03, fortnightly = 0.07, one to two times a week = 0.21, three to four times a week = 0.5, daily = 1.0, one to two times a day = 1.5.

Metric	Greens		Greens and green vegetables		Starchy tubers		Fruits		Total fruits and vegetables		Total daily junk food intake frequency
	Freq/day	g/day	Freq/day	g/day	Freq/day	g/day	Freq/day	g/day	Freq/day	g/day	
Mean	0.17	8.05	0.91	39.16	0.29	33.48	0.57	52.53	1.47	91.69	2.68
Q1	0.07	2.00	0.43	12.92	0.21	11.43	0.21	17.14	0.93	43.07	1.31
Median	0.21	6.43	1.03	30.50	0.21	17.14	0.50	40.00	1.43	77.41	2.3
Q3	0.21	12.86	1.21	42.86	0.50	40.00	1.00	80.00	2.07	114.02	3.67
IQR	0.14	10.86	0.79	29.94	0.29	28.57	0.79	62.86	1.14	70.95	2.36

**Table 4:** Actual intake of greens, vegetables (excluding tubers), and fruits against the WHO recommendations. *: Greens as per the Indian Council of Medical Research recommendation.

	Number (%) of students				
% intake of recommended values	<25%	25–49.9%	50–4.9%	75–99.9%	>100
Greens*	293 (74.4%)	82 (20.8)	9 (2.28)	3 (0.8)	7 (1.8)
Greens and green vegetables	328 (83.3)	40 (10.2)	12 (3.1)	5 (1.3)	9 (2.3)
Fruits	17 (4.3)	28 (7.1)	178 (45.2)	171 (43.4)	0
Total greens, vegetables, and fruits	251 (63.7)	108 (27.4)	21 (5.3)	7 (1.8)	7 (1.8)

**Figure 3:**
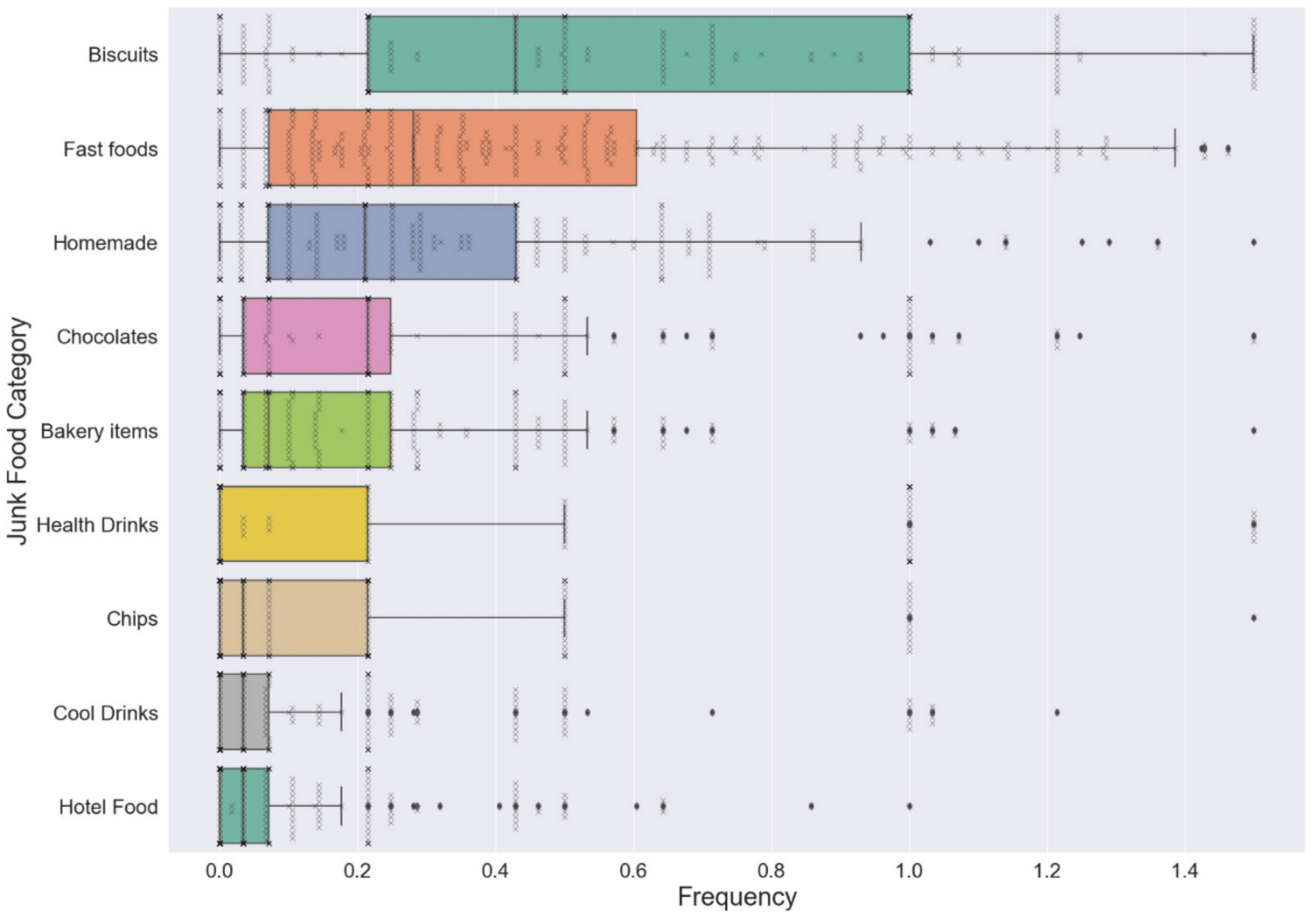
Most common junk foods consumed by students.

The median daily junk food intake frequency was two to three times per day (Table 3), which implied that the average child consumed junk foods at least two to three times a day. The frequency with which high-calorie, high-fat food items, such as biriyani and deep-fried items, were made at home was one to two times a week. Biscuits, homemade high-calorie foods, and so-called health drinks were among the most common junk food groups consumed (Figure 3).

Statistical analysis revealed that junk food intake was significantly associated with psychological distress (p < 0.001) with a small to medium effect size. Junk food also showed a significant association with significant GI symptoms (p = 0.008) with a small to medium effect size. However, when junk food was analyzed for association with the BMI of school students, the raw p-value showed significance, but on Bonferroni adjustment, it was not statistically significant. Similarly, total F&V intake showed a raw p-value of 0.04 when analyzed for association with psychological distress, but following Bonferroni correction, no significance was noted. No statistically significant association with either nutritional parameters or significant GI symptoms was noted for F&V intake (Table 5).

**Table 5:** Mann-Whitney U test results for dietary variables by psychological distress, gastrointestinal symptoms, and BMI category. Raw p-values shown; Bonferroni correction for m = 6 comparisons (family-wise α = 0.05/6 ≈ 0.008) determines adjusted significance (yes if raw p ≤ 0.008; no otherwise). A higher mean rank indicates higher values in that category. Effect size (r) calculated as |Z| / √N, where N = n1 + n2 (rank-biserial correlation; small effect ~0.1, medium ~0.3). SDQ = Strengths and Difficulties Questionnaire; BMI = body mass index

Dietary variable	Grouping variable	Category	n	Mean rank	Mann-Whitney U	Z	Raw p-value	Adjusted sig.	Effect size (r)
Frequency of Junk food per day	Psychological distress (SDQ)	No issues	230	135.89	4,690	-3.493	<0.001	Yes	0.206
		Psychological issues	58	178.64					
	Gastrointestinal symptoms	No significant symptoms	281	187.88	4,691	-2.645	0.008**	Yes	0.133
		Symptoms at least weekly for 2 or more months	113	221.43					
	BMI category	Abnormal BMI	149	209.38	4,692	-2.29	0.022*	No	0.117
		Normal BMI	236	182.66					
Green fruits and vegetables consumption (g/day)	Psychological distress (SDQ)	No issues	230	149.62	4,693	-2.078	0.038*	No	0.122
		Psychological issues	58	124.2					
	Gastrointestinal symptoms	No significant symptoms	281	193.88	4,694	-0.996	0.319	No	0.05
		Symptoms at least weekly for 2 or more months	113	206.51					
	BMI category	Abnormal BMI	149	192.41	4,695	-0.82	0.934	No	0.042

## Discussion

Our study revealed that significant GI morbidity, psychological issues, and abnormal nutritional indices exist in our student population and are increasing as compared to previous years. We also noted that F&V intake was very low and had decreased compared to previous reports, with junk food consumption being very high.

Of the 1,000 students invited to participate, 394 consented; this could have been due to health-conscious parents or more symptomatic children, who were willing to participate. The prevalence of significant GI symptoms was 28.7% in our students. Robin et al. [6] reported a 25% prevalence of significant GI issues as FGID, with a reported range between 9.9% and 27.5% in children and adolescents. As FGID is a diagnosis based on a symptom complex, the general reported prevalence is lower than what we noted; however, as our study involved individual GI symptoms, as opposed to FGID, we noted a higher prevalence of GI morbidity.

Psychological screening using the SDQ revealed 16.4% had definite and 18.6% had possible psychological issues. Keyho et al. [14] using the SDQ for screening reported 17.2% adolescents had definite and 28.8% had borderline abnormality in Nagaland, India, which was slightly higher than what we noted in Tamil Nadu. Malhotra et al., in a meta-analysis [15], reported 23% of schoolchildren in India had psychiatric disorders. However, we used only a screening tool and did not perform further detailed analysis to confirm psychological disorders.

More than one-third of the students (38.7%) had abnormal BMI, 19.5% had thinness, of which 8.3% had severe thinness, 13.7% were overweight, and 5.5%. were obese. A survey by the National Institute of Nutrition in 2012 [16] reported a prevalence of thinness in 35.8% children, of which severe thinness was 11.6% and overweight and obesity was 0.4%, using WHO recommendations. The Comprehensive National Nutrition Survey 2016-2018 [4] reported abnormal BMI in 34% children, with 27% thinness, and 7% overweight and obesity. Our data suggests an increasing trend of abnormal nutrition, with obesity increasingly replacing thinness in our population.

Nutritional parameters of schoolchildren are deteriorating compared to previous Indian studies, and overweight and obesity are increasing over thinness [4,16]. This is the scenario in most countries. UNICEF has reported an increase in abnormal nutrition from 2000 to 2016, with one-third of children being malnourished globally, overweight children increased from 10 to 20%, and 50% had hidden hunger [3]. We noted an even higher percentage with abnormal BMI of almost 40%.

The intake of greens and F&V was alarmingly low in terms of frequency and quantity. Median intake of greens was a mere 6.4 g/day at a median frequency of one to two times per week, against a recommended intake of 50 g/day. This was even lower than reported by other Indian researchers. Reported intakes ranged from 7 g/day in Andhra Pradesh to around 27 g/day in Himachal Pradesh [16,17]. As per the WHO, the minimum recommended intake of F&V is at least 350 g for children five to nine years old and 400 g for children above nine years old. Most countries recommend a far higher intake of F&V [18].

The recommended intake of vegetables for children aged five to nine years is 210 g, and for children more than nine years old, it is 240 g. However, the median intake of vegetables, including greens, was barely 30.16 g/day, with only 2.3% fulfilling the WHO recommendations. Further, 83.3% consumed less than 25% of the recommendation at a frequency of one to two times per day. Most Indian studies report very low intake of vegetables from 47.56 ± 3.35 to 140.5 g/day [19,20]. Choudary et al. [19] analyzed F&V consumption in an Indian population using the National Sample Survey data of 2011-2012 and reported a median per-capita/day consumption of vegetables of around 140 g. We noted an even more dismal intake of around 30 g/day in children. In 88% of the countries globally, Kalmpourtzidou et al. reported that the intake of vegetables is below WHO recommendations, and frequency is one to two per day [20]. WHO reports that 2% children eat vegetables fewer than once per day [3]. In our study, we noted that 45% of students took 50-75% of the recommended fruits daily, 43% consumed 75-100%, and none consumed 100%.

The median frequency of fruits was alternate days, and the median quantity was 40 g/day which is considerably below the standard age-appropriate recommendations. Peltzer and Pengpid in five Southeast Asian countries [21] reported that 28% children consumed fruits fewer than once a day. The National Nutrition Monitoring Bureau, India (NNMB) in 2012 [16] reported a mean intake of 22 g/day in 13-15-year-olds and 40 g/day in 10-12-year-olds, which is similar to our observations, whereas Choudhary et al. [19] reported a median per-capita intake of 50 g of fruit/day in adults.

The median frequency of F&V intake altogether was one to two times per day, and the median quantity consumed was 77.4 g/day, which is lower than that reported in similar Indian studies. Overall, 63.7% of students consumed less than 25% of the WHO recommendations, and only 1.8% consumed 100%. Peltzer and Pengpid [21], while evaluating 13-15-year-old school students, noted that Indian students had among the lowest intake, approximately 3.2 servings against a recommendation of five servings as per US norms.

Minocha et al. reported a per-capita F&V intake of 160 g/day in rural and 185 g/day in urban regions [22]. NNMB 2012 reported uniformly poor intake across all ages, with the mean intake of F&V between the ages of 7-15 being 67.8 g/day. As our students came from slightly higher socioeconomic strata, they might have had a slightly better F&V intake. Globally, countries also report poor F&V intake in adults and children. Lynch et al. [23] in their study among 11-year-olds from 10 developed countries in Europe documented that none of the countries had a mean intake of 400 g of F&V as per the WHO norms. Peltzer and Pengpid reported an F&V intake of less than 50% of the recommended and a mean intake of three servings per day in Southeast Asian countries, with India among the lowest intakes. Peltzer and Pengpid demonstrated poor intake of F&V in close to 30% of 13-15-year-old children in seven African nations [24].

The median frequency of daily junk food intake was 2.3, which implied that the frequency of intake of junk food was more than two times daily. This is considerably more than recommended by all countries, which is as minimal as possible. The Indian Academy of Pediatrics recommends one serving a week, consisting of less than 50% of the total daily recommended calories [25]. Gupta et al. reported that junk food contributed to 9.2% of calories and 20.9% of fat to the daily food intake [26].

Biscuits, “health drinks,” and “homemade junk” contributed most to high junk food intake. Traditional Indian dietary culture has worsened, and eating biscuits daily is acceptable to most caregivers, which begins as early as four months of age, as complementary food; awareness of its adverse effects is lacking. Most mothers are unaware of the adverse effects of ultraprocessed “health drinks” and are misled by advertisements, believing them to be beneficial to their children. Oily food is being cooked at home far more frequently than previously for its addictive taste and is replacing more healthy alternatives, such as F&V. The 2024 dietary guidelines from the ICMR [27] state that 56% of diseases in India are related to diet, and 34% of children five to nine years of age have high triglyceride levels. These alarming concerns should be addressed on a war footing to improve the diet and health of schoolchildren.

Statistical analysis (Table 5) revealed a significant association of junk food intake with significant GI symptoms and psychological morbidity, but not with nutritional abnormalities. What is equally important is that F&V intake was not statistically significantly associated with any of the abovementioned morbidities. Therefore, as per our data, there is a clear implication of junk food in GI morbidity and the psychological well-being of schoolchildren. The uniformly low intake of F&V may have limited the ability to detect a statistically significant protective association. An interesting theory is the “accumulation of risk theory,” which states that the protective value of F&V in adults is well established, but is not clearly established in children due to the accumulation of protective influence with increasing years of intake [28].

These findings highlight the need for targeted dietary interventions at multiple levels to reduce junk food intake and encourage the intake of F&V to improve overall gut health, psychological well-being, and nutritional status in school children.

To our knowledge, very few community-based studies are available in India that have explored the presence of significant GI symptoms in school-going children, though studies on specific GI diseases are available. Many GI morbidity-related studies are office-based and are prone to selection bias, abstraction bias, and ignore significant morbidity in the community. We included commonly consumed junk foods such as biscuits and so-called “health drinks,” especially homemade high-calorie foods, which have not been explicitly explored in many previous studies while evaluating junk food intake. Data collection by medics improved the quality of data.

However, being an observational study, we cannot ascribe causality, and being self-reported/parent-reported introduced subjectivity. Convenience sampling likely diminished generalizability. Selection bias is also possible with more participation from health-conscious parents or more symptomatic children. The differential collection of data for all three components of the proforma, namely, GI symptoms, dietary FFQ, and SDQ from parents for younger children and directly from older children, might have introduced heterogeneity. However, the number of parent-reported questionnaires, for GI symptoms, FFQ, and SDQ, was small. Data gathered from adolescents is prone to recall bias as well as social desirability bias, unlike the reporting by parents, which may not have similar bias. We did not adjust the statistical analysis for age, gender, and parental education.

In summary, GI morbidity is very commonly encountered in office practice. Globally, the intake of junk food is very high, and the intake of F&V is very low in children. This puts them at a very high risk of lifestyle diseases and morbidities in the future. We noted in our study that significant GI symptoms and morbidity are far more common in school-going children in the community than anticipated, and are only likely to increase due to the changing dietary pattern with steadily increasing junk food intake and declining F&V intake. This is also reflected in the trend of increasing obesity over thinness, which was predominant in the past in India. We also noted that psychological issues were not uncommon in schoolchildren due to these unhealthy dietary trends.

## Conclusions

Significant GI issues and nutritional and psychological morbidity were very high in schoolchildren. Junk food consumption has increased rapidly in recent years and has eroded F&V intake significantly. Junk food intake, GI symptoms, and psychological issues are strongly interlinked. An effort to inculcate the habit of increased F&V intake early on in life should be undertaken on a war footing. Common junk foods such as biscuits, health drinks, and homemade oily foods need to be curtailed urgently. Improved awareness of the benefits of F&V intake and the adverse effects of junk food on the body and mind needs to be imparted to parents, teachers, and students to achieve this goal.

## Appendices

Open the following link for the “Gastrointestinal Symptoms and Food Frequency Questionnaire”:

https://docs.google.com/spreadsheets/d/1sY2JqAZRjP9vPx-pNYSYPXUsP7gBMXNL/edit?usp=sharing&ouid=100192761133447934915&rtpof=true&sd=true
